# The relationship between increased body mass index and frailty on falls in community dwelling older adults

**DOI:** 10.1186/1471-2318-13-132

**Published:** 2013-12-06

**Authors:** Katie J Sheehan, Matthew DL O’Connell, Clodagh Cunningham, Lisa Crosby, Rose Anne Kenny

**Affiliations:** 1 Technology Research for Independent Living (TRIL), St.James’s Hospital, Dublin, Ireland; 2 Trinity College Institute of Neuroscience, Trinity College, Dublin, Ireland

## Abstract

**Background:**

The global population is becoming older and more overweight. The inter-relationship between frailty and falls is often seen in the older adult and is associated with poor health outcomes. Little is known about this relationship for those with excess body mass. This study aimed to assess the relationships between BMI, frailty and falls.

**Methods:**

Frailty, body mass index, clinical and demographic characteristics were assessed at baseline for 606 community dwelling adults aged 60 years and older. Falls were assessed prospectively with a semi-structured telephone interview two years later.

**Results:**

An increase in BMI contributed significantly to the identification of frail (Odds Ratio: 4.4; 95% Confidence Interval: 1.4, 13.6) older adults. A total of 346 falls by 148 participants were reported at follow up. Those with an increased BMI were significantly less likely to have experienced a fall between baseline and follow up assessments (p = 0.03). Despite these opposing trends a BMI greater than or equal to 30.0 kg.m^2^ did not alter the relationship between falls and frailty for the current cohort.

**Conclusions:**

This is the first study to assess the falls-frailty relationship for those with an increased BMI. Obesity was found to be protective against falling but not specifically in frail older adults.

## Background

With declining fertility and increasing life expectancy for most regions of the world, the global age structure has shifted from a younger to an older population [1]. From 1950 to 2000 the number of adults over the age of 60 increased by 401 million [1]. This trend is expected to continue with projected figures for 2050 of two billion adults over the age of 60 [1]. In Europe 66.1% of men and 53.8% of women over the age of 50 were recently reported as overweight or obese [2]. A 50% increase in the global proportion of adult obesity has been noted from 1980 to 2008 [3]. These figures suggest that the global population is becoming increasingly older and more overweight. This changing population demographic has undisputable implications with regard to health and economic costs.

Falls have been shown to result in increased morbidity and are responsible for over 17 million disability-adjusted life years lost [4]. One in three adults over the age of 65 will experience at least one fall each year [4]. A fall can have significant adverse outcomes including injury, hospitalisation, and admission to long term care, the development of fear of falling, activity restrictions, social isolation, reduced self-efficacy and quality of life [5]. Fried *et al.,*[6] described frailty as ‘…a clinical syndrome in which three or more of the following criteria were present: unintentional weight loss (10 lbs in past year), self-reported exhaustion, weakness (grip strength), slow walking speed, and low physical activity’. A history of falls in the older adult is regularly associated with the presence of frailty, whereby those classified as pre-frail/frail have a higher propensity to falling [7-10].

Recently a ‘U’ shaped correlation between obesity and frailty was reported, suggesting those who are underweight (body mass index (BMI) < 18.5) and those who are obese (BMI > 30) were more likely to present as frail [11]. This increased susceptibility towards frailty for obese older adults was surprising as frailty is often considered a wasting disorder [12]. Perhaps the association between obesity and frailty is the result of confounding, as obesity is often linked with slow walking speed, reduced physical activity levels, weakness and exhaustion [13-16]. Blaum *et al.,*[17] outlined several plausible physiological mediators of a relationship between frailty and obesity; these included the presence of an increased inflammatory marker C-reactive protein, and low antioxidant capacity with reduced carotenoids for their obese group.

Two prospective studies investigating the relationship between falls and obesity in the older adult have yielded conflicting results [18,19]. Himes & Reynolds [18] noted a relationship between falls and obesity, whereby the greater the extent of the obesity, the higher the falls risk. In contrast, Rosenblatt & Grabiner [19] reported similar prospective falls frequencies of 64.3% and 64.7% for obese and healthy weight older women respectively.

Perhaps the association between falls and obesity noted in the study by Himes & Reynolds [18] was mediated by an increased level of frailty for the obese older adults. No previous study has assessed the relationship between frailty and falls in the obese older adult. The current study had three aims:

1. To determine the cross sectional relationship between BMI and frailty in community dwelling older adults.

2. To determine the relationship between baseline frailty status and number of falls at two year follow up.

3. To determine if body mass index influences the relationship between falls and frailty in community dwelling older adults.

## Methods

A convenience sample from the Technology Research for Independent Living (TRIL) longitudinal study on ageing (http://www.trilcentre.org) was used for this research. Those community dwelling, ≥ 60 years of age, able to walk independently (with/without a walking aid), cognitively intact and able to provide informed consent were included in the TRIL cohort. Participants were recruited from St. James’s Hospital, or self-referred. Ethical approval was received from the St. James’s Hospital/Adelaide and Meath Hospital, incorporating the National Children’s Hospital Research Ethics Committee (approval reference number 2007/06/13). Participants completed an extensive baseline bio psychosocial assessment at the TRIL Clinic, St. James’s Hospital Dublin, Ireland. A semi-structured phone interview was conducted by a research nurse approximately two years following this assessment.

### Parameters

#### Demographic

Age, gender and social class were documented. Social class was determined using the Irish Central Statistics Office Census Social Class Classification (http://www.cso.ie). This seven point classification is based on a person’s occupation whereby professional workers are assigned a score of one, managerial and technical workers a score of two, non-manual workers three, skilled manual workers four, semi-skilled workers five, unskilled six, and all others gainfully occupied and unknown seven.

#### Clinical

Weight and height were measured according to standardised protocols to enable the calculation of BMI. International classification of BMI sub-groups were adopted whereby underweight was identified as a BMI of < 18.5 kg.m^2^, healthy weight as a BMI of 18.5 to <25 kg.m^2^, overweight as a BMI of 25 to <30 kg.m^2^, and obese a BMI of >30 kg.m^2^[3]. Polypharmacy was defined as the regular use of four or more prescription medications [20]. The presence of co-morbidities associated with falls and frailty were documented during the baseline comprehensive assessment, and were confirmed with hospital medical records. These included osteoarthritis, Parkinson’s disease, diabetes, congestive cardiac failure, chronic obstructive pulmonary disease, peripheral vascular disease, cancer, hypertension, and atrial fibrillation.

#### Frailty

Frailty status was determined by five criteria closest to those outlined by Fried *et al.,*[6,21]:

I. Exhaustion: Present for those who answered ‘yes’ to ‘In the last week, did you feel in at least three days that everything you did was an effort?’ and/or ‘ In the last week did you feel in at least three days that you could not get going?’.

II. Weakness: Present for those in the lowest 20^th^ percentile of grip strength stratified by gender and quartiles of BMI.

III. Gait velocity: Present for those in the lowest 20^th^ percentile of gait velocity stratified by gender as measured by the GAITRite™ walkway system.

IV. Physical activity: Present for those in the lowest 20^th^ percentile of the number of hours spent walking outdoors per week stratified by gender.

V. Weight loss: Present for those who reported at least 1 kg of unintentional weight loss in the three months prior to their assessment.

Those who met none of the criteria were classified as robust, those who met 1–2 criteria pre-frail and those who met 3–5 criteria frail.

### Falls

Falling was defined as a sudden, unintentional change in position causing an individual to land on a lower level, on an object, the floor, the ground or other surface [22]. Participants were contacted two years after the baseline assessment and an interview to determine the number of falls incurred was conducted. Participants who experienced one fall in the follow up period were classified as fallers. Participants who reported two or more falls in the follow-up period were classified as recurrent fallers.

### Statistical analyses

Demographic and clinical characteristics, frailty proportions and indicators were expressed in terms of mean and standard deviations, ratios, and positive sample proportions for the total sample and for the sample according to follow-up faller status. Statistical tests for trends associated with faller status were computed using logistic regression for categorical variables.

Multi-nominal logistic regression was employed to determine the cross sectional relationship between BMI and frailty, and the association between baseline frailty status and falls at follow up. Analyses were adjusted for potential demographic and clinical confounders. Finally a multi-nominal logistic regression between baseline frailty and falls at follow-up stratified by BMI was completed. Alpha was set at 0.05 for all analyses.

## Results

Six hundred and six participants completed the baseline assessment. Of the 606 participants contacted at follow-up 85 did not complete the telephone interview for the following reasons: 30 were deceased, five were admitted to long term care, nine had no recall of the baseline assessment and were deemed unable to give informed consent, 29 declined, and 12 could not be contacted. At follow up 546 participants were included in the analysis. Those who were not followed up were significantly older, frailer and demonstrated a higher proportion of polypharmacy, Parkinson’s disease, chronic obstructive pulmonary disease, peripheral vascular disease, cancer and atrial fibrillation (p ≤ 0.05). 13 participants were classified as underweight (BMI < 18.5kg.m^2^), due to the small sample and the current focus on those with a higher BMI they were excluded from the regression analyses.

Baseline demographics, clinical measures, frailty proportions and frailty indicators for the total sample and the sample stratified by faller status at follow up are presented in Table 1. Those who reported falling at the follow up interview were older at baseline (p = 0.005). In addition, those with polypharmacy (p <0.001), Parkinson’s disease (p = 0.002), congestive cardiac failure (p = 0.03) or peripheral vascular disease (p = 0.04) were significantly more likely to have experienced a fall between the baseline and follow up assessments. A decreasing trend in BMI was noted for an increase in reported falls, however this trend did not reach significance (p = 0.08).

**Table 1:** Baseline demographics, clinical measures and frailty indicators for the total sample and the sample stratified by faller status at follow up

			**Faller status at follow up**		
	**All (n = 606)**	**Not followed up (n = 85)**	**Non faller (n = 373)**	**Faller (n = 85)**	**Recurrent faller (n = 63)**
Demographic					
Age (years) mean(SD) ^†^	72.8 (7.2)	75.6 (8.3)	71.7(6.6)	71.6(7.1)	74.8(7.3)
Gender (m:f)	189:416	30:55	124:243	16:74	18:46
Social class mean(SD)	3.3 (1.7)	3.6 (1.8)	3.2(1.6)	3.2(1.7)	3.3(1.6)
Clinical					
BMI (kg.m^2^) mean (SD)	26.8 (4.6)	26.0 (6.1)	27.2(4.5)	26.5(5.2)	26.2(4.0)
Polypharmacy %	48.8	56.5	40.9	50.0	70.3
Osteoarthritis %	62.0	69.9	60.8	66.7	60.9
Parkinson’s %^†^	4.8	5.9	2.7	4.4	12.5
Diabetes %	8.3	9.4	7.9	6.7	4.7
Congestive cardiac failure %^‡^	31.2	38.8	27.8	28.9	39.1
Chronic obstructive pulmonary disease %	11.2	21.2	10.4	5.6	9.4
Peripheral vascular disease %^‡^	4.8	14.1	2.7	3.3	7.8
Cancer %	3.5	4.7	3.0	3.3	1.6
Hypertension %	44.4	49.4	40.9	46.7	45.3
Atrial fibrillation %	4.3	5.9	4.4	5.6	3.1
Frailty %					
Robust	47.5	30.6	54.0	50.0	31.2
Pre-frail	43.2	47.1	41.1	43.3	56.2
Frail	8.1	22.4	4.4	6.7	10.9
Frailty Indicators %					
Weight loss %	14.9	15.3	4.1	5.5	10.9
Exhaustion %^†^	22.3	30.6	18.3	16.7	32.8
Weakness %^†^	36.0	49.4	30.0	38.9	43.8
Slow walking velocity %^‡^	10.2	22.4	7.4	6.7	18.8
Decreased activity levels %	13.7	25.9	9.5	16.7	10.9

SD = standard deviation; ^†^ p ≤ 0.01; ^‡^ p ≤ 0.05.

Faller indicated one fall event, recurrent faller indicates two or more falls in the follow up period.

**Figure 1:**
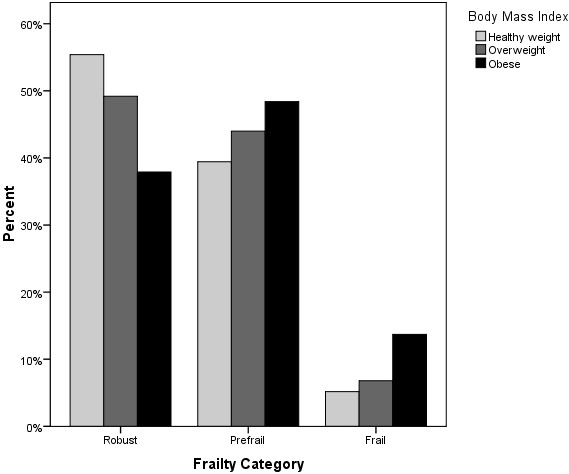
Frailty frequencies classified by body mass index.

At baseline 47.5% of the sample was classified as robust, 43.2% as pre-frail and 8.1% as frail. Figure 1 outlines the proportions of those classified as robust, pre-frail and frail according to BMI category. Table 2 describes the proportion of obese, overweight and healthy weight participants who met each of the frailty criteria. Those with obesity were significantly more likely to meet exhaustion, weakness, reduced gait velocity and decreased activity levels frailty criteria (p ≤ 0.05). Those of a healthy weight were more likely to meet the weight loss frailty criteria (p = 0.03).

**Table 2:** Cross tabulations between BMI category, and frailty criteria

	**BMI**		
	**18.5 to <25 kg.m**^**2 **^**(n = 606)**	**25 to <30 kg.m**^**2 **^**(n = 254)**	**>30 kg.m**^**2 **^**(n = 124)**
Frailty Indicators %			
Weight loss %^‡^	19.3	11.6	12.4
Exhaustion %^‡^	19.2	21.3	29.0
Weakness %*	28.9	33.5	51.6
Reduced walking velocity %^†^	7.0	9.8	14.5
Decreased activity levels %*	9.3	12.2	23.4

* p ≤ 0.001; ^†^ p ≤ 0.01; ^‡^ p ≤ 0.05.

Table 3 provides the results of the multi-nominal logistic regression for frailty measures at baseline. A BMI ≥ 30 kg.m^2^ contributed significantly to the identification of pre-frail (Odds Ratio: 2.5; 95% CI: 1.5, 4.2) and frail (Odds Ratio: 8.3; 95% CI: 3.3, 21.0) older adults. This finding persisted even after adjusting for potential demographic and clinical confounders. A BMI of 25.0-29.9 kg.m^2^ was associated with pre-frailty (Odds Ratio: 1.5; 95% CI: 1.0, 2.3) and frailty (Odds Ratio: 2.2; 95% CI: 1.0, 5.1) in unadjusted models, however with the inclusion of demographic and clinical characteristics these relationships were no longer statistically significant. Older age contributed, to a lesser extent, to the identification of pre-frailty (Odds Ratio 1.1, 95% CI 1.0, 1.1) and frailty (Odds Ratio 1.2, 95% CI 1.1, 1.2) for the current cohort.

**Table 3:** Multinominal logistic regression assessing the relationship between BMI, pre-frailty and frailty

	**Pre-frailty**		**Frailty**	
	**Model 1**	**Model 2**	**Model 1**	**Model 2**
	**Odds ratio (95**% **confidence interval)**			
BMI ≥30.0 kg.m^2^	2.5(1.5, 4.2)*	2.1(1.2, 3.7)^†^	8.3(3.3, 21.0)*	4.4(1.4, 13.6)^†^
BMI 25.0-29.9 kg.m^2^	1.5(1.0, 2.3)^‡^	1.4(1.0, 2.2)	2.2(1.0, 5.1)^‡^	1.7(0.6, 4.7)
Age	1.1(1.1, 1.1)*	1.1(1.0, 1.1)*	1.2(1.2, 1.3)*	1.2(1.1, 1.2)*
Gender	0.7(0.4, 1.0)^‡^	0.5(0.3, 0.8)^‡^	0.5(0.3, 1.1)	0.2(0.1, 0.8)^‡^
Social class		1.3(1.1, 1.4)*		1.1(0.8, 1.4)
Polypharmacy		0.9(0.6, 1.4)		0.6(0.2, 1.7)
Atrial fibrillation		0.5(0.2, 1.5)		0.7(0.1, 4.2)
Hypertension		1.1(0.7, 1.7)		1.9(0.8, 4.6)
Arthritis		0.9(0.6, 1.3)		0.6(0.2, 1.6)
Parkinson’s disease		0.3(0.1, 0.9)^‡^		0.2(0.0, 1.2)
Congestive cardiac failure		0.6(0.4, 0.9)^‡^		0.1(0.1,0.4)*
Peripheral vascular disease		0.3(0.1, 0.9)^‡^		0.1(0.0, 0.6)^†^
Diabetes		0.5(0.2, 1.1)		0.3(0.1, 1.2)
Cancer		0.6(0.2, 1.8)		0.1(0.0, 0.5)
Chronic obstructive pulmonary disease		0.8(0.4, 1.5)		0.3(0.1, 0.7)

Model 1 includes only BMI, age and gender (referent category = female); models 2 additionally includes potential clinical and demographic confounders. * p ≤ 0.001; ^†^ p ≤ 0.01; ^‡^ p ≤ 0.05.

Pre-frailty was identified by the presence of 1–2 frailty indicators. Frailty was identified by the presence of 3–5 frailty indicators.

A total of 346 falls by 148 participants were reported at the follow up phone interview. 85 experienced a single fall, and 63 experienced multiple falls. A positive faller status at follow up was significantly associated with the frailty indicators weakness (p = 0.02), reduced gait velocity (p = 0.02) and exhaustion (p = 0.03) (Table 1). Baseline frailty status was a significant predictor of recurrent faller (Odds Ratio: 1.4; 95% CI: 1.1, 1.6) status at follow-up assessment. With adjustment for social class, polypharmacy, atrial fibrillation, hypertension, congestive cardiac failure, diabetes, chronic obstructive pulmonary disease, peripheral vascular disease, osteoarthritis, cancer and Parkinson’s disease the relationship between recurrent falls and frailty was marginally reduced (Odds Ratio: 1.3; 95% CI: 0.8, 1.6).

A negative trend for the report of falling between the two assessments was noted for higher levels of BMI. At least one fall was reported by 29.2% of those of a health weight, 27% of those classified as overweight, and 18% of those classified as obese. This trend was significant for fallers between the two assessments (p = 0.03). The trend was non-significant for those who experienced recurrent falls between the assessments (p = 0.2). Table 4 details an analysis of the relationship between falls and frailty stratified by BMI category. The relationship between recurrent falls and frailty was significant only for those with a BMI of 25.0-29.9 kg.m^2^ (Odds Ratio: 3.2; 95% CI: 1.5, 6.7).Within this group frailty was associated with a decreased likelihood of reporting a single fall (Odds ratio 0.4; 95%CI: 0.2, 0.9). Overall, the stratified analyses did not show consistent trends towards a different relationship between falls and frailty across levels of BMI.

**Table 4:** Multinominal logistic regression for the relationship between falls and frailty stratified by BMI

		**Faller**	**Recurrent faller**
		**Odds ratio (95**% **Confidence interval)**	
Total sample	Frailty	1.1(0.7, 1.4)	1.4(1.1, 1.6)*
	Age	1.0(1.0, 1.0)	1.1(1.0, 1.1) ^†^
	Gender	0.4(0.2, 0.8)*	0.8 (0.4, 1.5)
BMI ≥30.0 kg.m^2^	Frailty	1.7(0.6, 4.7)	0.6(0.2, 2.1)
	Age	0.9(0.8, 1.0) ^‡^	1.1(1.0, 1.3)
	Gender	0.5(0.1, 2.6)	0.2(0.1, 3.4)
BMI 25.0-29.9 kg.m^2^	Frailty	0.4(0.2,0.9) ^‡^	3.2(1.5,6.7) ^†^
	Age	1.0(1.0,1.1)	1.0(1.0,1.1)
	Gender	1.2(0.6,2.3)	0.4(0.2,0.9)
BMI 18.5-24.99 kg.m^2^	Frailty	1.2(0.6,2.5)	1.8(0.8, 4.1)
	Age	1.0(1.0, 1.1)	1.0(1.0,1.1)
	Gender	0.5(0.2,1.3)	1.8(0.8. 4.6)

* p ≤ 0.001; ^†^ p ≤ 0.01; ^‡^ p ≤ 0.05.

Gender referent category = female.

## Discussion

This is the first study to assess the falls-frailty relationship stratified by BMI. Obesity was inversely related to falls and was positively associated with frailty, but from our data did not seem to modify the relationship between falls and frailty. Obesity was found to be protective against falling but not specifically in frailer older adults.

With increased BMI, an increase in the sample proportions of the frailty criteria exhaustion, weakness, reduced walking velocity, and decreased activity levels were noted. A negative association between BMI and weight loss was found. For the current study BMI demonstrated a strong positive association with frailty status at baseline. The relationship persisted after adjustment for potential demographic and clinical confounders. This is in contrast to previous research which describes a ‘U’ shaped relationship whereby those with a BMI of 25.0-29.9 kg.m^2^ were less likely to present as frail than their healthy weight or obese peers [17,23]. Age, gender, congestive cardiac failure and peripheral vascular disease were also significantly associated with frailty for the current cohort.

Fried’s weight loss criterion is believed to reflect wasting in older adults [6]. For the current study it was noted that an increased BMI was negatively associated with weight loss. This would suggest that those with a higher BMI are less susceptible to wasting, as defined by weight loss. However sarcopenia has been shown to affect older adults independent of their BMI [23,24]. It may be hypothesised that ‘true’ wasting, sarcopenia or muscle loss, may be better reflected by the weakness criterion for those with an increased BMI.

Previous studies on the relationship between BMI and falling indicate that increased BMI increases the risk of falls [18,25] or adds no additional risk of falls [19]. The current study found that for an increase in BMI there was in fact a reduced risk of falling for older adults. Sharkey *et al.*, [26] noted that for older adults with severe obesity (BMI ≥ 35kg.m^2^) both static and dynamic stability were impaired. Overweight and obese older adults have been shown to adopt a more tentative gait pattern, with a slower walking velocity and an increased base of support [13]. As a result of these adaptations, stability is in fact improved [27]. These alterations may inadvertently represent a protective effect against falls. In addition those with obesity were more likely to meet the reduced physical activity frailty criterion. This would suggest that adults with obesity present with less opportunity to fall as they are less physically active.

Overweight and obese participants were identified as more likely to be frail, but less likely to experience a fall. This was surprising given the general consensus of the association between falls and frailty in older adults [7-10]. The results of the multinominal logistic regression analyses stratified by BMI indicated that BMI did not clearly influence the falls-frailty relationship for the current cohort. The positive relationship between frailty and falling was evident for the full sample. In the stratified analyses, the relationship between frailty and recurrent falling was strongest in the overweight group, however frailty was inversely associated with single falls in this group. Similarly there was a clear, if non-significant, positive relationship between frailty and single falls in normal weight participants combined with a trend towards a negative relationship with recurrent falls. The lack of coherent pattern across categories suggests these apparent differences maybe be more likely to be due to chance differences in the number of falls in each group, rather than a clear effect of BMI on the relationship between frailty and falling in this sample.

There are some limitations to this study. Frailty was not longitudinally assessed, and therefore the temporal relationship between changes in frailty and BMI cannot be determined. Falls history was recorded at a two year follow up assessment. This history was self-reported and may have led to an underreporting of falls and an underestimation of the effects presented here. There are many definitions of frailty. For the present study we chose to focus on physical frailty, as defined by the frailty phenotype. While this model is widely accepted, it allows only a broad categorisation of levels of frailty in comparison to the finer gradation of individual risk possible with deficit accumulation models [28]. For the stratified analyses the relatively small sample sizes and number of falls within each subgroup may have limited the ability to detect effects.

## Conclusion

Frailty is traditionally perceived as a wasting disorder associated with an increased risk of falls, morbidity, hospitalisation, and mortality [29]. This study aimed to assess the falls-frailty relationship for adults with increased BMI. It was found that those with an increased BMI were more likely to present as frail and less likely to fall. Despite these opposing trends a BMI ≥30.0 kg.m^2^ did not clearly alter the relationship between falls and frailty in the current cohort.

## Competing interests

The authors declare that they have no competing interests.

## Authors’ contributions

KS conceived the study, performed the statistical analyses, and drafted the manuscript. MDLOC performed statistical analysis and contributed to the drafting of the manuscript. CC and LC recruited all participants and completed the assessment. RAK participated in the design of the study and helped to draft the manuscript. All authors read and approved the final manuscript.

## Pre-publication history

The pre-publication history for this paper can be accessed here:

http://www.biomedcentral.com/1471-2318/13/132/prepub
